# Effect of Hand and Ultrasonic Scaling-Root Planing Methods on Tooth Surface Topography: An In-Vitro Atomic Force Microscopy Study

**DOI:** 10.7759/cureus.46925

**Published:** 2023-10-12

**Authors:** Neebha Kumari, Lynn Johnson, Hemlata Yadav, Arindam Das, Brajendra Umrao, Radhika Gera

**Affiliations:** 1 Department of Periodontology, Rama Dental College, Kanpur, IND

**Keywords:** root planing, hand scaling, atomic force microscopy, ultrasonic unit, surface roughness

## Abstract

Purpose: Any instrumentation on the tooth surface for plaque or calculus removal will cause some amount of roughness on the tooth surface. Hence, this study was proposed to investigate the effects of hand and ultrasonic scaling and root planing on enamel and cementum, respectively.

Materials and methods: Forty tooth samples were prepared from extracted maxillary and mandibular first pre-molars and were divided randomly into four groups of 10 samples each. Group l: received ultrasonic scaling on enamel; Group II: received hand scaling on enamel surface; Group III: root planing with an ultrasonic unit on the cementum samples; and Group IV: root planing using hand curettes on cementum surface. The amount of roughness produced on the surface of each sample of all four groups was evaluated using atomic force microscopy (AFM) and statistically analyzed using Chi-square, ANOVA, and Wilcoxon tests.

Results: The results suggested that the surface roughness produced on both crown and root after scaling and root planing (SRP) using a hand instrument is lower than that of an ultrasonic unit. The roughness of the crown was found to be lower than that of the root after SRP using both a hand instrument and an ultrasonic unit and was also statistically significant (P = 0.034). In contrast, there is not enough evidence to conclude a significant difference (P=0.13) between root planing using hand instruments and ultrasonic scaler groups. The combined p-value using the Chi-square test (P=0.026) suggests a statistically significant overall difference between crown and root groups.

Conclusion: From the present study, the authors concluded that scaling as well as root planing using an ultrasonic unit cause more tooth (enamel and cementum) surface roughness as compared to hand scaling and root planing. While there is no significant difference in the surface roughness of root-cementum produced due to the root planing in both groups, crown-enamel exhibits a significant difference after scaling in both groups.

Clinical significance of the study: Rough, uneven tooth surfaces negatively influence the anticipated healing of the periodontium by providing retention areas for microbial dental plaque.

## Introduction

The basic goal of periodontal therapy is to eliminate or reduce the contributing risk factors and microbial ecology of periodontitis, thereby halting further development of the disease and thus preserving the overall health, comfort, function, and aesthetics of the dentition and prevention of its recurrence. According to the proposal laid by the American Academy of Periodontology (AAP), any procedure aimed at maintaining the health of the periodontium should be carried out using minimally invasive techniques [1], including scaling, root planing, and at-home and professional oral hygiene procedures [2]. The most regularly performed procedure for treating periodontal disease, i.e., scaling and root planing (SRP), is regarded as the “gold standard” therapy in comparison to other therapeutical procedures [3].

Scaling-root planing is carried out either with hand or ultrasonic instruments. The advantages of hand instrumentation over machine scaling include better supervision of the instrumentation procedure and an enhanced tactile response to the operator. But, the procedure is cumbersome, exhausting, and requires experience to develop the proper skills, whereas ultrasonic scalers permit easy access to deep pockets and furcation areas and are more time-competent and less exhausting for dentists [4]. However, it is reported that this procedure causes a roughened tooth surface, a procedure that has multifactorial influences, including procedure time, angulation, and pressure of the instruments. A positive correlation between the rate of plaque deposition (both supragingival and subgingival) and surface roughness has been noted [5]. Thus, the aim of this study was to compare the tooth surface roughness (enamel and cementum) produced after scaling and root planing either by hand or ultrasonic instrumentation using atomic force microscopy (AFM) on prepared tooth enamel and cementum samples.

## Materials and methods

Study design

Forty tooth sections of 2x4x1mm dimensions were obtained from the crown and root portions of freshly extracted intact maxillary and mandibular first pre-molars removed due to orthodontic reasons and were randomly divided into four groups of 10 samples each: Group I: 10 samples of crown-enamel treated with an ultrasonic scaler; Group II: 10 samples of crown-enamel treated with hand scalers; Group III: 10 samples of root-cementum treated with ultrasonic root planing tips; and Group IV: 10 samples of root cementum treated with universal curettes. 

Study settings

Hand scaling was done on 10 enamel samples using Hu-Friedy’s sickle scaler U15/30 and root planing on 10 cementum samples by Hu-Friedy’s Columbia Universal Curettes #2R/2L. Machine scaling and root planing were done on 10 enamel and 10 cementum samples using an ultrasonic unit (Woodpecker UDS-J) with its recommended scaling (G4) and root planing (P3) tips for the respective groups with a medium (level-4) power setting and water coolant. The procedure for all four groups was done by activating the respective instrument at a 60-700 working angle with 20 overlapping scaling or root planing strokes. After SRP was performed on all 40 samples, these were subjected to atomic force microscopy (AMF, Asylum Research, MFP-3D) to measure the surface roughness caused by each procedure, and images were recorded. The complete procedure, from sample preparation to SRP, was performed at the Department of Periodontology, Rama Dental College, UP, by one investigator (a post-graduate student) to reduce operator-induced procedural bias.

Statistical analysis

After obtaining the required data and images from the topographic analysis by AFM, these were compared using Chi-square, Anova, and Wilcoxon tests to statistically analyze the surface roughness difference in all the groups. A p-value less than or equal to 0.05 was considered statistically significant. Minitab and R-project software were used to record and analyze the data.

Ethical consideration

Ethical approval was obtained from the ethical committee of the institution with institutional review board number (IRB) IEC/RDC/2023/11.

## Results

The combined p-value using the Chi-square test (P=0.026) suggests a statistically significant overall difference between crown and root groups, and the p-value when using Anova and Wilcoxon was nearly the same for the present analysis (Tables 1, 2).

**Table 1:** Amount of surface roughness (in nm) produced in each group

*Groups*	*Count*	*Sum*	*Average*	*Variance*	*Standard deviation*
*Group I-Ultrasonic scaling*	10	571.166	57.117	799.693	28.279
*Group II-Hand scaling*	10	321.436	32.144	389.973	19.748
*Group III-Ultrasonic root planing*	10	950.533	95.053	1313.874	36.247
*Group IV- Root planing by hand instrument*	10	680.644	68.064	1584.096	39.801

**Table 2:** Statistical analysis of surface roughness of roots and crowns using ANOVA one-way, Wilcoxon, and Chi-squared tests SS: Sum of squares, MS: Mean sum of squares, F: Anova coefficient

Crown – Enamel					
Source of Variation	SS	F	F crit	P-value (ANOVA)	P- value (Wilcoxon)
Between Groups	3118.254	5.242	4.414	0.0343	0.0288
Within Groups	10706.99				
Total	13825.24				
Root – Cementum					
Source of Variation	SS	F	F crit	P-value (ANOVA)	P- value (Wilcoxon)
Between Groups	3642.004	2.513	4.414	0.1302	0.1431
Within Groups	26081.73				
Total	29723.74				

Evaluation of crown-enamel surface roughness: The average surface roughness value observed in Group I (ultrasonic scaling) is 57.117 ± 28.279 nm, and in Group II (hand scaling), it is 32.144 ± 19.748 nm, indicating that ultrasonic scaling causes more surface roughness as compared to hand scaling. The p-value of 0.034 (which is smaller than 0.05) indicates a statistically significant difference between hand scaling and ultrasonic scaling groups.

**Figure 1:**
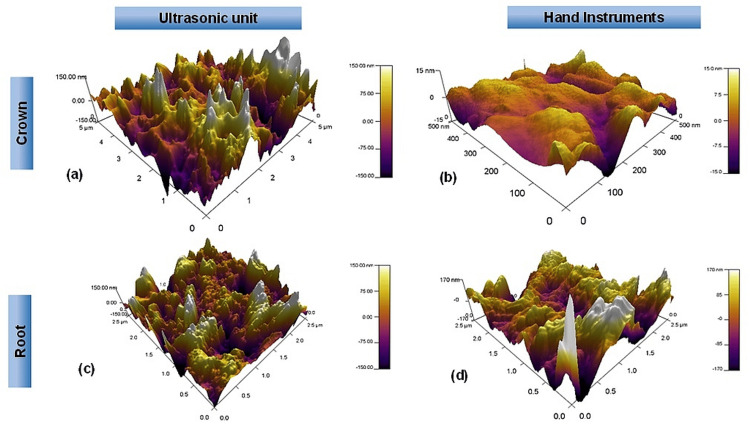
AFM images of the surface roughness produced in the four groups after scaling and root planing by hand instruments and an ultrasonic unit (a) ultrasonic scaling in crown-enamel samples, (b) hand-scaling in crown-enamel samples, (c) ultrasonic root planing in root-cementum samples, (d) root planing using hand instrument in root-cementum samples

**Figure 2:**
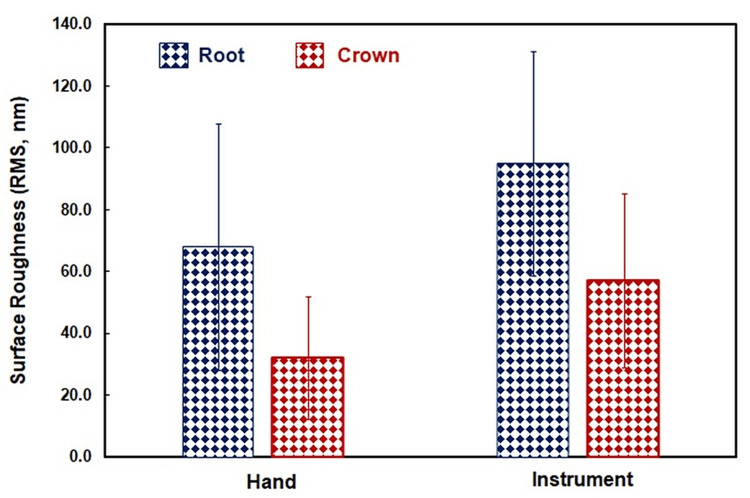
Plot showing the amount of surface roughness produced in the four groups after scaling and root planing by hand instruments and an ultrasonic unit

Evaluation of root-cementum surface roughness: The average surface roughness values observed in Group III (ultrasonic root planing) are 95.053 ± 36.247 nm and in Group IV (hand instruments) are 68.064 ± 39.801 nm, stating that ultrasonic root planing causes more surface roughness as compared to curettes. The p-value of 0.130 (which is larger than 0.05) indicates that there is no statistically significant difference between the two groups for root planing (Figures 1, 2).

## Discussion

Nowadays, as periodontal disease is a common finding in the general population, many receive oral hygiene maintenance treatment, mainly scaling and root planing, at regular intervals [6]. Elimination of supra as well as subgingival plaque-calculus, besides a decline in the inflammatory level of the disease, is the main key feature of periodontal therapy. Though scaling-root planing should ideally eliminate plaque, stains of extrinsic origin, calculus, and bacterial components, numerous in vitro data have confirmed iatrogenic effects following instrumentation by specialists [7-9]. These surface lesions and irregularities increase the surface area of the tooth, thus promoting bacterial colonization and plaque formation and, thereby, compromising daily plaque removal by the patients [10]. This surface roughness also depends on many factors, such as the type of prophylactic method used, the number of treatments undergone till date, the angulation of the hand instrument or the scaling tip, the shape of the instrument used, lateral pressure, etc. [11]. Although many studies have aimed to define the critical level of hard tooth tissues’ roughness, this value remains undetermined. However, it is widely accepted that the threshold surface roughness for plaque retention is 0.2 µm (200nm) [12,13].

The micro-roughness of natural tooth enamel, as obtained from various studies, ranges from 0.59-0.66μm (590-660nm) [14]. This uneven enamel surface inhibits proper cleaning, leading to bacterial accumulation, plaque formation, tooth staining, and poor esthetics. Restoring the enamel to its original morphology after any prophylactic procedure is still a challenge for all clinicians and researchers. Similarly, loss of cementum should ideally remain confined to the 3-7 μm layer of endotoxin invasion in addition to the biofilm. The cemental thickness is known to vary with tooth types, root area, and patient age [15]. Generally speaking, the thickness of cementum is considered to be 250μm. It is apparent from these figures that any excessive instrumentation can quickly reduce or completely remove the cementum layer, leading to poor healing [16]. Removal of excessive cementum may cause exposure of dentinal tubules, leading to root sensitivity [17]. It is predicted that ultrasonic scalers cause 6.3-55.9μm and curettes 100μm of root surface roughness after root planing [18]. A lack of common opinion concerning the effect of diverse instrumentation on the crown and root surfaces of a tooth is still in the literature. While some studies believed that manual instrumentation led to excess root surface removal, other researchers showed ultrasonic scalers to produce the same deleterious effect [19]. As practitioners and patients opt for treatment procedures that are completed in less time, ultrasonic systems are preferred over hand instruments as they shorten the SRP interval. However, as tooth sensitivity and abrasion after oral prophylaxis are a concern for a few patients, it is crucial for both the dentist and the patient to decide the best treatment option that causes the least roughness to the tooth surface during SRP.

The present in vitro study compared the surface roughness produced by ultrasonic units and hand instruments on enamel and cementum surfaces. The topographic study of the AFM showed that ultrasonic scaling and root planing tips produced higher surface roughness than hand instruments, as shown in the AFM microphotographs. To our knowledge, this is the first AFM study done to compare ultrasonic units and hand instruments on tooth enamel and cemental surfaces for studying the tooth surface roughness produced by them. Hence, the results were compared with studies using other techniques for analyzing tooth surface roughness. The results were similar to the study done by Yildirim et al., who concluded that the surface roughness produced by the ultrasonic scaler on the enamel and cementum surfaces was higher than that of manual instruments [20]. These authors suggested that hand instruments such as Gracey curettes aided enhanced instrument control and tactile proprioception during instrumentation, whereas the decreased tactile sensation and vibrational forces of the ultrasonic unit induced more surface roughness following root planing [20]. Aspriello et al. also observed uneven, roughened tooth surfaces and grooves post-ultrasonic instrumentation compared to hand instrumentation [21]. Similarly, Meyer et al., in their in-vitro study on surface roughness using SEM, stated that the hand curette formed the least roughness, followed by the Roto-Pro instrument, while the ultrasonic curette and the diamond produced the most irregular surfaces [7]. Kerry, too, in her study on extracted teeth, stated that ultrasonic instrumentation created significantly rougher root surfaces in comparison to hand curettes [22].

Our study has some limitations that can be overcome in future research. The study was conducted on a small sample size using a single technique for surface roughness evaluation. The study can be done using larger samples and multiple methods for evaluating surface roughness, such as confocal microscopy, histological analysis, etc. Moreover, as the study was in vitro, the outcome of this study cannot be applied directly to clinical situations. Factors such as the composition of the saliva, the patient’s oral hygiene, temperature, and pH can also change the outcome of the study. Future studies involving patients must be carried out to cross-check our results and their implementation in clinical settings.

## Conclusions

The conclusion drawn by the authors, indicating that both scaling and root planing with an ultrasonic unit result in increased surface roughness compared to hand instrumentation, raises important considerations for clinical practice. It suggests that clinicians need to carefully weigh the benefits and drawbacks of each technique based on the specific needs of their patients. While hand instrumentation may preserve a smoother root surface, it requires a higher level of manual dexterity and may be more time-consuming. In contrast, ultrasonic units offer efficiency and accessibility in removing deposits, which can be advantageous in cases where rapid and thorough cleaning is required. Therefore, the choice between these techniques should be made on a case-by-case basis, considering factors such as the patient's oral health condition, the specific treatment goals, and the clinician's proficiency with the chosen method. 
